# BAR502, a dual FXR and GPBAR1 agonist, promotes browning of white adipose tissue and reverses liver steatosis and fibrosis

**DOI:** 10.1038/srep42801

**Published:** 2017-02-16

**Authors:** Adriana Carino, Sabrina Cipriani, Silvia Marchianò, Michele Biagioli, Chiara Santorelli, Annibale Donini, Angela Zampella, Maria Chiara Monti, Stefano Fiorucci

**Affiliations:** 1 University of Perugia, Department Surgical and Biomedical Sciences, Perugia, Italy; 2 University of Perugia, Department of Medicine, Perugia, Italy; 3 University of Naples Federico II, Department of Pharmacy, Naples, Italy; 4 University of Salerno, Department of Pharmacy, Fisciano, Salerno, Italy

## Abstract

Non-alcoholic steatohepatitis (NASH) is a highly prevalent chronic liver disease. Here, we have investigated whether BAR502, a non-bile acid, steroidal dual ligand for FXR and GPBAR1, reverses steato-hepatitis in mice fed a high fat diet (HFD) and fructose. After 9 week, mice on HFD gained ≈30% of b.w (P < 0.01 versus naïve) and were insulin resistant. These overweighting and insulin resistant mice were randomized to receive HFD or HFD in combination with BAR502. After 18 weeks, HFD mice developed NASH like features with severe steato-hepatitis and fibrosis, increased hepatic content of triacylglycerol and cholesterol and expression of SREPB1c, FAS, ApoC2, PPARα and γ, α-SMA, α1 collagen and MCP1 mRNAs. Treatment with BAR502 caused a ≈10% reduction of b.w., increased insulin sensitivity and circulating levels of HDL, while reduced steatosis, inflammatory and fibrosis scores and liver expression of SREPB1c, FAS, PPARγ, CD36 and CYP7A1 mRNA. BAR502 increased the expression of SHP and ABCG5 in the liver and SHP, FGF15 and GLP1 in intestine. BAR502 promoted the browning of epWAT and reduced liver fibrosis induced by CCl_4_. In summary, BAR502, a dual FXR and GPBAR1 agonist, protects against liver damage caused by HFD by promoting the browning of adipose tissue.

Non alcoholic fatty liver disease (NAFLD) and steato-hepatitis (NASH) are a highly prevalent human disorders for which no approved treatment is currently available^1^. Thus, while several experimental approaches are under development, NASH remains a largely un-meet need^2^,^3^. NASH occurrence is highly correlated with obesity, insulin resistance, and dyslipidemia and while patients with simple steatosis have a good prognosis, the overall morbidity and mortality are increased massively in patients with NASH due to increased risk for cardiovascular complications, cirrhosis and hepatocellular carcinoma^4^,^5^. The pathogenesis of NASH is multifactorial and triggered by environmental factors such as hypernutrition in the context of a genetic predisposition but also requires a yet poorly-defined second hits. Insulin resistance and visceral adipose tissue inflammation are thought to be central in the pathogenesis of NAFLD and especially NASH^6^,^7^,^8^,^9^,^10^. Several rodent models of NAFLD and NASH are available, but the relevance of these models to the human NASH is imperfect showing substantial heterogeneity of gene and pathway regulation in comparison to human NASH, reflecting the diversity of pathways that can lead to steatosis^11^,^12^. Among the murine models, steatohepatitis induced by long-term administration of a high fat diet (HFD) and fructose leading to steatosis, inflammation and fibrosis, shows the better correlation to human NAFLD and NASH in comparison with other murine models of fatty liver disease^12^,^13^.

Bile acids are amphipatic molecules synthesized in the liver from oxidation of cholesterol. Beside their role in nutrient absorption, primary bile acids, chenodeoxycholic acid (CDCA) and cholic acid (CA), and secondary bile acids, deoxycholic acid (DCA) and lithocholic acid (LCA), and their glycine and taurine conjugates, are signaling molecules exerting a variety of regulatory function by activating a family of cell-surface and nuclear receptors collectively known as the “bile acid activated receptors” (BARs)^14^. The best characterized members of the BARs family are the G-protein coupled receptor GPBAR1 (also known as TGR5) and the farnesoid-x-receptor (FXR). GPBAR1 and FXR are highly expressed in entero-hepatic tissues where their activation regulates a number of metabolic functions^2^,^14^,^15^.

We have previously shown that 6-ECDCA, also known as obeticholic acid, a dual FXR and GPBAR1 ligand, attenuates liver steatosis that develop in *ApoE−/−* mice and Zucker rats^16^,^17^. Additionally FXR ligands have been shown effective in reducing liver steatohepatitis (but not fibrosis) in patients with NAFLD and NASH^18^,^19^. The use of obeticholic acid, however, causes itching (75% of patients with primary biliary cholangitis), suggesting that additional approaches need to be develop to treat the full spectrum of NASH patients^18^.

The 6α-ethyl-3α, 7α-dihydroxy-24-*nor*-5β-cholan-23-ol (NorECDCOH christened BAR502), a recently discovered dual FXR and GPBAR1 ligand, is a non-bile acid steroidal dual GPBAR1 and FXR ligand with a preferential activity toward GPBAR1^20^. In the present study we demonstrate that BAR502 cures metabolic features, liver steato-hepatitis and fibrosis caused by exposing mice to a HFD and fructose by promoting the browning of adipose tissue. Present results support the notion that dual FXR and GPBAR1 ligands could be effective in treating human NASH.

## Materials and methods

### Chemicals

Design and synthesis of BAR502 (6α-ethyl-3α, 7α-dihydroxy-24-*nor*-5β-cholan-23-ol) has been described previously^20^. The agent was dissolved daily in drinking water containing 1% methyl cellulose before its administration by gavage (100 μl).

### Animal model of NASH

C57BL6 mice 24 weeks old were fed a high fat diet containing 60% kj fat (ssniff ^®^ EF acc. D12492 (I) mod.) and fructose in drinking water (42 g/l) or normal diet (6 mice) for 18 weeks^11^,^12^,^13^. After 10 weeks of HFD, mice were randomized to receive HFD alone (9 mice) or HFD plus BAR502 (15 mg/kg/day) body weight by gavage (9 mice) for 8 weeks. HFD mice were administered 100 μl of 1% methyl cellulose in drinking water. Mice were housed under controlled temperatures (22 °C) and photoperiods (12:12-hour light/dark cycle), allowed unrestricted access to standard mouse chow and tap water and allowed to acclimate to these conditions for at least 5 days before inclusion in an experiment. The care and use of the animals were approved by the Institutional Animal Care and Use Committee of the University of Perugia and were in accordance to European guidelines for care of experimental animals. Protocols were approved by the Italian Minister of Health and Istituto Superiore di Sanità (Italy) and were in agreement with the European guideline for use of experimental animals (permission n. 41/2014-B released to AD). The general health of the animals was monitored daily by the Veterinarian in the animal facility. The study protocol caused minor suffering, however, animals that lost more than 25% of the initial body weight were euthanized. At the day of sacrifice mice were deeply anesthetized with a mixture of tiletamine hypochoride and zolazepam hypocloride/xylazine at a dose of 50/5 mg/Kg. Food intake was estimated as the difference of weight between the offered and the remnant amount of food at 7-days intervals. The food was provided as pressed pellets and the residual spillage was not considered.

### Liver fibrosis

Liver fibrosis was induced by carbon tetrachloride (CCl_4_) administration according to a previously published method^21^. For this purpose, C57BL6 mice were administered i.p. 500 μL/Kg body weight of CCl_4_ in an equal volume of paraffin oil twice a week for 8 weeks. CCl_4_ mice were randomized to receive BAR502 (15 mg/Kg daily by gavage) or vehicle (distilled water).

### Tissue histology

For histological examination, portions of the right and left liver lobes and epididymal white adipose tissue (epWAT) were fixed in 10% formalin, embedded in paraffin, sectioned and stained with Sirius red and Hematoxylin/Eosin (H&E), for morphometric analysis. NASH severity was scored in H&E-stained cross sections using an adapted grading system of human NASH as described previously^6^,^22^. The level of macrovesicular and microvesicular steatosis was determined at 40 × magnification relative to the total liver area analyzed and expressed as a percentage. Inflammation was scored by counting the number of aggregates of inflammatory cells per field using a 100 × magnification. The average of five random fields was taken. Relative values against the average of the HFC control group were calculated. Hepatic fibrosis was identified using Sirius Red stained slides and evaluated using an adapted grading system of human NASH as described previously. The presence of pathological collagen deposition was scored as either absent (0), observed within perisinusoidal/perivenular or periportal area (1), within both perisinusoidal and periportal areas (2), bridging fibrosis (3) or cirrhosis (4). The sum of the scores (degree of steatosis, hepatocyte ballooning, lobular inflammation, and portal inflammation) was considered as the total pathology score.

### Biochemical analyses

AST, triglyceride, total- and HDL- cholesterol, albumin and fasting glucose concentrations were quantified using an automated clinical chemistry analyzer (Cobas, Roche) with enzymatic methods. Plasma insulin levels were measured by ELISA assays according to the manufacturer’s instructions. The homeostatic model assessment (HOMA) of IR and insulin sensitivity index were calculated by the concentrations of plasma glucose and insulin.

### Hepatic lipid analysis

For determination of total triglyceride and cholesterol content, liver samples from left hepatic lobules of ∼100 mg of liver were homogenized with 1 ml of T-PER (Pierce). The homogenates were used for protein concentration analysis (Bradford assay, Bio-rad, Milan, Italy), and 100 μl of tissue extracts added to 1.6 ml CHCl_3_: methanol (2:1) for 16 h at 4 °C, after which 200 μL of 0.6% NaCl was added and the solution centrifuged at 2,000 *g* for 20 min. The organic layer was removed and dried by Speed Vac System (HETO-Holten, Waltham, MA). The resulting pellet was dissolved in 100 μL phosphate buffered saline containing 1% Triton X-100 and triglyceride, cholesterol, and FFA content was measured by specific enzymatic reagents.

### OGTT and ITT

After 9, 13 and 18 weeks of HFD administration, the mice were fasted overnight and orally administered glucose (1.5 g/kg body weight) for OGTT or fasted for 4 h and intraperitoneally injected insulin (0.35 unit/kg body weight) for ITT. The blood glucose concentrations were measured at 0, 15, 30, 60, 90, and 120 min after feeding or injection using a portable glucose meter (Accu-Check Go, Roche). Plasma insulin levels were measured by Mercodia Ultrasensitive Mouse Insulin ELISA assays according to the manufacturer’s instructions.

### Tissue Microarray

Total RNA was extracted from liver, epWAT, BAT, gastrocnemius and intestine (4 mice/group) using TRIzol reagent (Invitrogen) and reverse transcribed with SuperScript II Reverse Transcriptase (Invitrogen) following the manufacturer’s instructions. A total of 10 ng of cDNA was pipetted into each well of a 96-well Custom RT^2^ Profiler PCR ArrayCAPM13349, RT^2^ Profiler PCR Array, Qiagen (http: www.qiagen.com/it/products/catalog/assay-technologies/real-time-pcr-and-rt-pcr-reagents/rt2-qpcr-primer-assays/l) and amplified following the manufacturer’s instructions in StepOnePlus instrument (Applied Biosystems). This RT^2^ PCR array is designed to assess a total of 75 genes:30 genes for liver, 6 for gastrocnemius, 6 for intestine, 22 for epWAT and 11 for BAT; 2 housekeeping genes were used for each tissue for data normalization. The RT^2^ Profiler PCR Array also includes control elements for: Genomic DNA contamination detection, RNA sample quality and General PCR performance. Data analysis is based on the ΔΔC_T_ method with normalization of the raw data to either housekeeping genes.

### Quantitative Real-Time PCR analysis

RNA extracted from liver, epWAT, BAT, gastrocnemius and intestine was subjected to reverse transcription; for Real Time PCR 10 ng cDNA were amplified in a 20 μL solution containing 200 nM of each primer and 10 μL of 2X SYBR FAST Universal ready mix (Invitrogen). All reactions were performed in triplicate, and the thermal cycling conditions were as follows: 10 min at 95 °C, followed by 40 cycles of 95 °C for 10 sec, 55 °C for 10 s and 60 °C for 30 sec in StepOnePlus instrument (Applied Biosystems). The relative mRNA expression was calculated and expressed as 2^−(ΔΔCt)^. Expression of the respective genes was normalized to both B2M and GAPDH mRNA as an internal controls. PCR primer sequences were:mouse-GAPDH: ctgagtatgtcgtggagtctac and gttggtggtgcaggatgcattg; mouse-B2M: ctttctggtgcttgtctcactg and ttcagcatttggatttcaatgt; mouse-ATG5: gcatcaagttcagctcttcctt and gcatcagcttctttcatacac; mouse-ATG7: tttctgtcacggttcgataatg and gtgaatccttctcgctcgtact; mouse-ATG12: gctgaaggctgtaggagacact and gagttccaacttcttggtctgg; mouse-LC3a: ctgtaaggaggtgcagcagat and cttgaccaactcgctcatgtta; mouse-adiponectin: gacaggagatcttggaatgaca and gaatgggtacattgggaacagt; mouse-UCP1: ctcactcaggattgtgcctctac and tctgaccttcacgacctctgta; mouse-PPARα: cagaggtccgattcttccac and gatcagcatcccgtctttgt; mouse-Rantes: ctgctgctttgcctacctct and tccttcgagtgacaaacacg ; mouse- IL6: cttcacaagtcggaggctta and ttctgcaagtgcatcatcgt; mouse PPARγ: gccagtttcgatccgtagaa and aatccttggccctctgagat ; mouse aP2: aaacaccgagatttccttcaaa and tcacgcctttcataacacattc; mouse-UCP3: ccaacatcacaagaaatgc and tacaaacatcatcacgttcc.

### Immunohistochemistry

Immunohistochemistry on white adipose tissue and on brown adipose tissue (used as positive control) were performed. Formalin-fixed paraffin-embedded sections (7 μm thick) were deparaffinized and then antigen retrieval was performed by treatment with protease. Sections were then washed in PBS. and treated with hydrogen peroxide (3%) for 5 minutes to block endogenous peroxidase. Non specific binding was blocked by treatment with BSA (Bovine Serum Albumin) 8% plus Triton 0,2% in PBS for 30 minutes. Anti UCP-1 (Abcam 1:500) in PBS (2% BSA +0,05% Triton) was incubated overnight at +4 °C. After washing, anti-rabbit IgG (1:200 Vector) in PBS was incubated for 1 hour, section were washed and incubated with ABC (Vectastain ABC Kit elite) and then, after washing with DAB peroxidase substrate kit (Vector), according to the manufacturer instructions. Sections were counterstained with haematoxylin, dehydrated and coverslips mounted with mounting medium.

### Cell culture and cell differentiation

3T3-L1 preadipocytes were plated at 37.5 × 10^3^ cells/well in 24-well plates. Preadipocytes were grown to confluence in DMEM and 10% FCS^23^. Two days after reaching confluence (day 0), the cells were stimulated with differentiation mix (Mix) alone (DMEM, 10% FCS, 517 μM 3-isobutyl-1-methylxanthine, 1 μM dexamethasone, and 172 nM insulin). At day 2, Mix was replaced by insulin medium (DMEM, 10% FBS, and 172 nM insulin) alone. At day 4 cells were stimulated 24 hours with 10 μM BAR 502 or 10 μM BAR501.

### Western Blotting

Total lysates were prepared by solubilization of 3T3L1 cells in E1A lysis buffer (250 mM NaCl, 50 mM Hepes pH 7.0, 0.1% NP40, 50 mM EDTA) containing phosphatase and protease inhibitors. Protein extracts were electrophoresed, blotted to nitrocellulose membrane and then incubated with primary antibodies against UCP-1 (Abcam) and Tubulin (Sigma). Primary antibodies were detected with the horseradish peroxidase (HRP)-labeled secondary antibodies. Proteins were visualized by SuperSignal West Dura Extended Duration Substrate (Thermo Fisher Scientific Inc.) according to the manufacturer’s instructions.

### Bile acids determination

Bile acids pools were measured by liquid chromatography-tandem mass spectrometry (MS/MS) analysis, using chromatographic conditions as described elsewhere^24^. The stock solutions of the individual tauro-conjugated and un-conjugated bile acids were prepared separately in methanol at a concentration of 1 mg/mL. All stock solutions were stored at −20 °C. Calibration standards were prepared by combining appropriate volumes of each bile acid stock solution and methanol. The calibration range was from 10 nM to 100 μM of each bile acid in the final solution. Mice serum sample aliquots of 20 μL were mixed with 80 μL of CH_3_OH, shaken continuously, vortexed and, after centrifugation at 16000 g for 10 min, the clear supernatant was transferred to a new vial, snap frozen and lyophilized. The sample was then re-dissolved in 40% water: 60% MeOH with 0.1% formic acid and ammonium acetate 5 mM. A bile acids extraction yield of 95% has been measured using bile acids standards addition in plasma sample before and after deproteinization procedure.

For gallbladder and fecal samples, 10 mg of lyophilized gallbladder or feces was manually pestled using a mortar and dissolved in 1 mL CH_3_OH. After centrifugation at 16000 g for 10 min, 500 μL of supernatants were lyophilized and reconstituted in 100 μl of 40% water: 60% MeOH with 0.1% formic acid and ammonium acetate 5 mM.

### Liquid chromatography and mass spectrometry

For LC–MS/MS analysis, chromatographic separation was carried out on the HPLC–MS system Q-TRAP 6500 LC-MS/MS System from AB Sciex equipped with Shimadzu LC-20A LC and Auto Sampler system. The mixture was separated on a Synergi Fusion RP 4 μ 80°A from Phenomenex (150 × 2.00 mm). Tauro-conjugated and non-conjugated bile acids were separated at a flow rate of 200 μl/min using a methanol–aqueous ammonium acetate (NH_4_OAc) gradient. Mobile phase A was water containing 5 mM ammonium acetate and 0.1% formic acid, mobile phase B was methanol, containing ammonium acetate at 5 mM and 0.1% formic acid. The gradient started at 65% B and increased to 85% B in 23 min, kept at 85% B for 5 min then decreased to 65% B in 1 min and kept at 65% B for 10 min. ESI was performed in negative ion mode, the ion source temperature was set at 280 °C. The tune page parameters were automatically optimized injecting taurocholic acid at 1 μM as standard.

### Statistical analysis

All of the data are shown as the means ± SEM. Difference among group means were estimated using one-way ANOVA followed by Tukey’s post hoc test by GraphPad Prism 5.0 software. Significance was set up at *p* < 0.05.

## Results

### BAR502 protects from development of steato-hepatitis and fibrosis in dietary model of NASH

**Figure 1:**
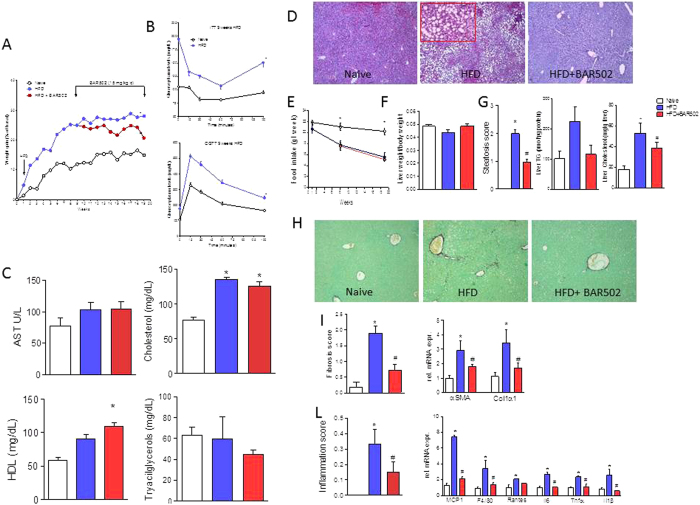
BAR502 reduced body weight gain and reduced hepatic lipid partition in mice fed HFD. Mice were fed a HFD and fructose for 18 weeks. BAR502 was administered at the dose of 15 mg/kg/day starting on day 63 (week 9). The data shown are (**A**) body weight (% delta weight); (**B**) glycemic response to oral glucose tolerance test (OGTT) and to insulin-tolerance test (ITT) (both after 9 weeks of HFD). (**C**) plasma levels of AST, cholesterol, HDL and triacylglycerols measured at the end of the study. The data shown in Panels (**A**–**C)**, are mean ± SE of 9 mice. (**D**) Hematoxylin and eosin (H&E) staining on mice liver tissues showing severe steatosis and ballooning of hepatocytes in mice feed a HFD for 19 weeks. These changes were significantly attenuated by treating the HFD mice with BAR502. The data are mean ± SE of 9 mice. (**E**) Food intake through the study expressed as mg/week/mouse. The data shown in Panels A–C, are mean ± SE of 9 mice. The data show that while HFD mice had reduced food intake in comparison to mice on standard chow diet, BAR502 did not reduced food intake. Panels F–L. Impact of BAR502 on (**F**) liver weight, (**G**) steatosis (Steatosis score) and liver TG and Cholesterol content. (**H**) Sirius red staining of liver sections; (**I**) fibrosis score and hepatic expression of αSMA and COL1α1 mRNA; (**L**) inflammation score and hepatic expression of pro-inflammatory genes. F4/80 is a markers for macrophages. Results are the mean ± SE of 5–9 mice per group. *p < 0.05 versus naive mice, ^#^p < 0.05 versus HFD mice. Values are normalized to B2M and ACTβ, the relative mRNA expression is expressed as 2^(−ΔΔCt).^

To induce NASH-like features, C57BL6 mice were fed a HFD and fructose (collectively HFD) for 18 weeks. HFD mice gained significantly more weight than naïve mice starting from week 2. After 4 weeks of HFD food intake in this group started to decrease and the trend was maintained throughout the study (Fig. 1A,B). After 9 weeks, mice feed the HFD were significantly more heavy (Fig. 1A) than naïve mice (≈30% increase of body weight compared to 10%, *P* < 0.01), had higher glucose plasma levels and were insulin resistant as demonstrated by glucose response to OGTT and ITT (Fig. 1B, upper and lower panel; P < 0.05 versus naïve mice).

HFD, insulin-resistant, mice were than randomized into two groups: HFD alone or HFD in combination with BAR502, 15 mg/kg/day, and followed for additionally 9 weeks (see above for the protocol). At the end of the study, HFD mice developed biochemical and histopathology features of NASH, including elevated AST, cholesterol and HDL plasma levels (Fig. 1C; *P* < 0.01), while plasma triacylglycerol levels were similar to that of naïve mice.

While the liver weight and the percent of liver weight to the body weight did not changed in this dietary model in comparison with naïve mice of the same age (Fig. 1F), the histopathology analysis, the liver of HFD mice showed evidence of NASH-like features, including severe steatohepatitis with extensive lobular fat deposition, diffuse hepatocytes ballooning and microvescicolar steatosis (Fig. 1D, middle panel and inset). Mice on HFD had significantly higher steatosis scores and increased liver content of triacylglycerol and cholesterol than control mice (Fig. 1G, P < 0.01 versus naïve mice). Additionally, HFD mice developed moderate liver fibrosis as measured by morphometric analysis of fibrotic areas in liver sections immune-stained with Sirius red and by assessment of the expression of αSMA and α1-Collagen, two pro-fibrogenetic genes (Fig. 1H and I; P < 0.01 versus naïve). Finally, feeding a HFD caused an influx of inflammatory cells as assessed by measuring the inflammatory score at histopathology and expression of F4/80 (a marker for macrophages) along with IL-1β, IL-6, MCP-1, RANTES and TNFα mRNA (Fig. 1L, P < 0.05 versus naïve).

**Figure 2:**
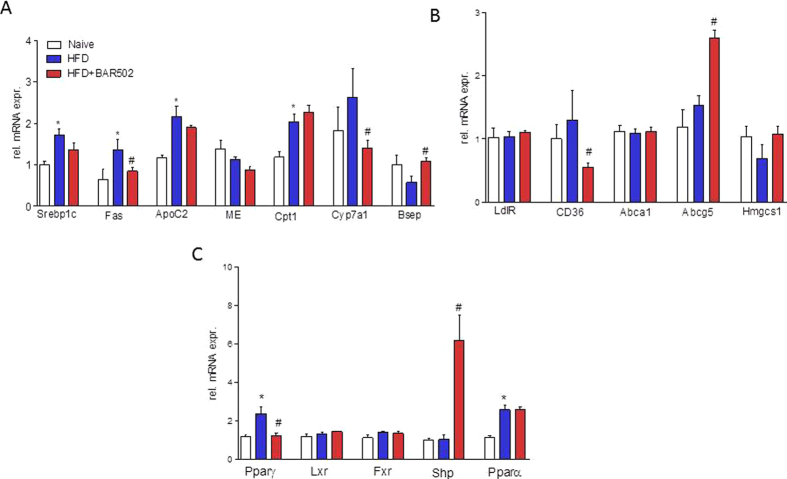
BAR502 represses fatty acid synthesis, activates export of lipids from hepatocytes and activates FXR target genes in the liver. Relative hepatic mRNA expression of genes involved in (**A**) Triacylglycerols and fatty acid metabolism, (**B**) Cholesterol metabolism, and (**C**) Nuclear receptors. Results are the mean ± SE of 5 mice per group. *p < 0.05 versus naive mice, ^#^p < 0.05 versus HFD mice. Values are normalized to B2M and ACTβ, the relative mRNA expression is expressed as 2^(−ΔΔCt)^.

As shown in Fig. 2, eating of a HFD increased the expression of genes encoding for proteins involved in regulating cholesterol and triacylglycerol metabolism: Srepb1c, Fas ApoC2, Cpt1, CD36 and Pparα and γ (Fig. 2A–C), while the expression of other nuclear receptors such as Lxr, Fxr and Shp was unchanged.

These NASH-like features were reversed by feeding mice with BAR502. As detailed above, treatment with BAR502 was initiated after 9 week of HFD, i.e. at a time point at which mice had reach a stable body weight and were insulin resistant. Thus, from week 9 to week 18 mice administered the HFD alone gained an additional 3% of body weight, while treating mice with BAR502 resulted in ≈10% decrease of body weight (P < 0.05 versus HFD). These change associates with robust remodeling of plasma lipid metabolism profile, since BAR502 increased HDL (Fig. 1C). Administering HFD mice with BAR502 reversed liver lipid accumulation resulting in a robust reduction of liver steatosis score and along with reduction of liver lipid content (Fig. 1D–G; P < 0.05 versus HFD). In addition, BAR502 reduced liver fibrosis scores by 70% and expression of pro-fibrogenetic genes (αSMA and α1-collagen), as well as the inflammatory score and liver expression of F4/80, IL-1β, IL-6, MCP-1, RANTES and TNFα mRNA (Fig. 1H–L).

These phenotypic changes associate with remodeling of the liver expression of genes involved in lipid metabolism. As illustrated in Fig. 2A–C, BAR502 effectively reduced the relative expression of Fas and Srepb1c mRNA (n = 5, p < 0.05). Quantitation of Cyp7α1 and Bsep mRNA levels confirmed that BAR502 *in vivo* behaves as full FXR agonist as it reduced Cyp7α1 while induced Bsep and Shp (Fig. 2, n = 5, p < 0.05).

Amelioration of hepatic steatosis by BAR502 was also linked to the modulation of uptake and exportation of cholesterol from the liver, as demonstrated by the investigation of genes involved in cholesterol metabolism. Indeed, BAR502 significantly reduced the mRNA level of CD36 and upregulated the expression of ABCG5, a membrane transporter involved in cholesterol efflux from hepatocytes (Fig. 2; n = 5, p < 0.05). BAR502 reduced Pparγ mRNA (Fig. 2C; n = 5, p < 0.05).

### BAR502 causes the browning of epWAT

**Figure 3:**
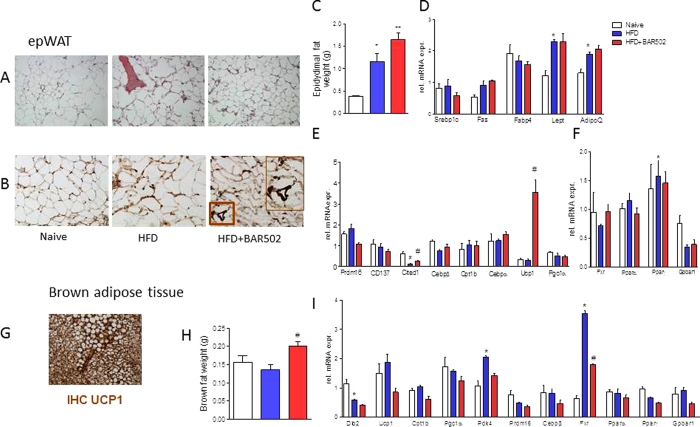
BAR502 improved morphometry and promotes the browning of epWAT of C57BL6 fed HFD mice. (**A**) H&E staining on mice epWAT tissues. BAR502 reduced the size of adipocytes and reduced the extent of inflammatory infiltration; Magnification in 20X; (**B**) Immunohistochemistry analysis of the expression of uncoupling protein 1 (UCP1) in epWAT of naïve mice and mice on HFD with or without BAR502. Magnification is 20x. Inset: enlargement of the area enclosed by square showing staining of UCP1. Magnification is 40X (**C**) Effect of BAR502 on epWAT weight. Changes in mRNA expression of epWAT genes. Data shown are relative expression of genes involved in: (**D**) adipogenesis and fatty acid transport and metabolism; (**E**) brite/beige trans-differentiation and (**F**) nuclear receptors. (**G**) The expression of UCP1 in brown adipose tissue of C57BL6 mice was assayed by immunohistochemistry and used as positive control. (**H**) Effect of BAR502 on BAT weight. (**I**) Changes in mRNA expression of BAT marker genes and nuclear receptors. Results are the mean ± SE of 5 mice per group. *p < 0.05 versus naive mice, ^#^p < 0.05 versus HFD mice. Values are normalized to B2M and ACTβ, the relative mRNA expression is expressed as 2^(−ΔΔCt)^.

The above mentioned changes demonstrate that feeding mice with BAR502 reshapes liver lipid partitioning in this dietary model of NASH. Because the WAT is the major storage tissue for fat, we have then examined whether BAR502 modulates fat depots in the model. Eating a HFD for 18 weeks increased of weight of epWAT by 3-fold (P < 0.01) while the BAT weight remained unchanged (Fig. 3C and H). BAR502 caused a further increase in epWAT weight but it also increased the amount of BAT (Fig. 3A–C and H; P < 0.05 versus HFD). These changes associate with the browning of epWAT as assessed by morphometry and gene array (3B). Exposure to the high fat diet increased the mRNA levels of Fas, leptin and adiponectin (Fig. 3D–F, n = 5, p < 0.05). In contrast, exposure to BAR502 reduced the size of adipocytes and effectively induced the expression of Ucp1, a marker of brite/beige trans-differentiation, as assessed by RT-PCR and immune-histochemistry (Fig. 3B and E) and expression of Cited1 mRNA (Fig. 3E, n = 5, p < 0.05). As illustrated in Fig. 3G, the BAT expresses very high levels of UCP1 at immune-histochemistry and treating mice with BAR502 increased the weight of BAT (Fig. 3H), but had no effect on the level of major genes involved in lipid metabolism in this tissue, including relative expression of Ucp1 mRNA (Fig. 3I, n = 5, p < 0.05).

### BAR502 regulates browning of fat depot in the muscles

**Figure 4:**
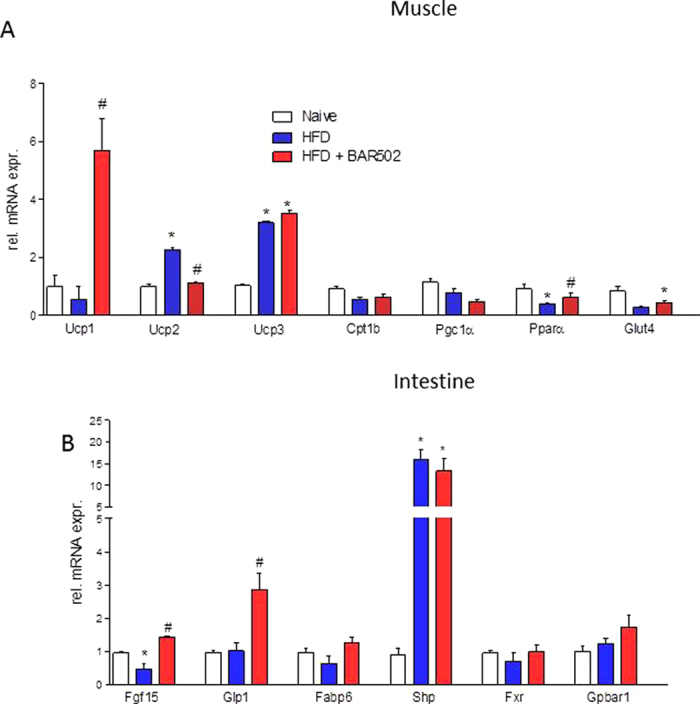
Effects of BAR502 on muscle and intestine. Change in transcript levels of genes involved in regulating: (**A**) muscular energy metabolism, and (**B**) enteric metabolism. Results are the mean ± SE of 5 mice per group. *p < 0.05 versus naive mice, #p < 0.05 versus HFD mice. Values are normalized to B2M and ACTβ, the relative mRNA expression is expressed as 2^(−ΔΔCt)^.

Administering HFD mice with BAR502 also resulted in a 2–6 folds induction of Ucp1, 2 and 3 mRNA in muscles, further highlighting that BAR502 has the ability to redirect the metabolism of lipid in the whole body (Fig. 4). Additionally, BAR502 increased the expression of Fgf-15 and Shp (two FXR target genes), and Glp1 (a GPBAR1 target gene) in the intestine (P < 0.05 versus HFD) demonstrating that this agent acts as a dual FXR and GPBAR1 ligand *in vivo*.

### BAR502 increases the insulin sensitivity

**Figure 5:**
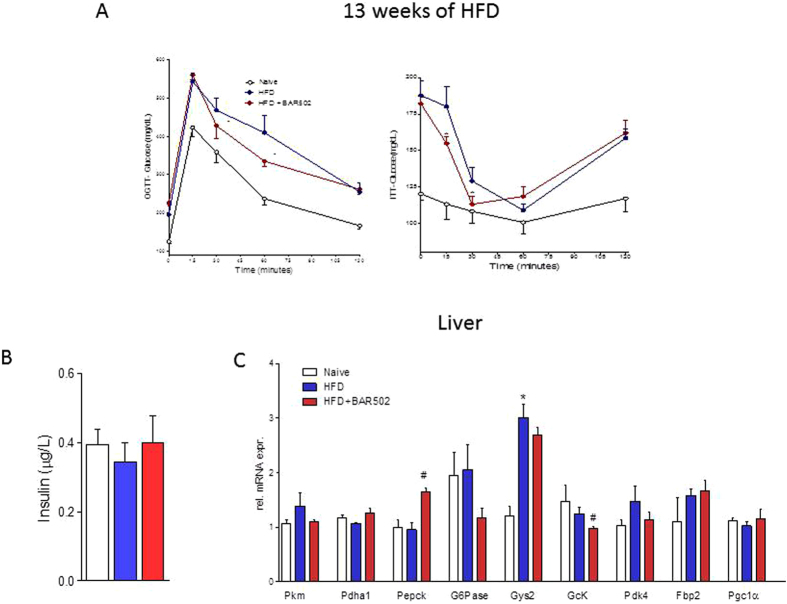
Effects of BAR502 on glucose and insulin sensitivity. The data shown are the glycemic response to (**A**) oral glucose tolerance test (OGTT) and to insulin-tolerance test (ITT) (after 13 weeks of HFD). Mice were fasted by were assuming fructose in drinking water. (**B**) Insulin plasma levels measure after 18 weeks of HFD. (**C**). Liver expression of genes involved in glucose metabolism. Results are the mean ± SE of 5 mice per group. *p < 0.05 versus naive mice, #p < 0.05 versus HFD mice. Values are normalized to B2M and ACTβ, the relative mRNA expression is expressed as 2^(−ΔΔCt)^.

Treating mice with BAR502 increased insulin sensitivity as measured by assessing the OGTT and ITT after 4 weeks administration, as shown in Fig. 5A (P < 0.05 versus HFD). The beneficial effect of BAR502 on OGTT was maintained up to the end of the study (data not shown). However, BAR502 had a minor impact on insulin plasma levels measured at the end of the study (Fig. 5B, n = 9) and liver expression of genes involved in glucose metabolism (Fig. 5C, n = 5; *P < 0.05 versus naïve; #P < 0.05 versus HFD). This could partially due to fructose feeding that was continued through the whole duration of the study including the day of sacrifice.

### BAR502 inhibits the synthesis of endogenous bile acids in a dietary model of NASH

**Figure 6:**
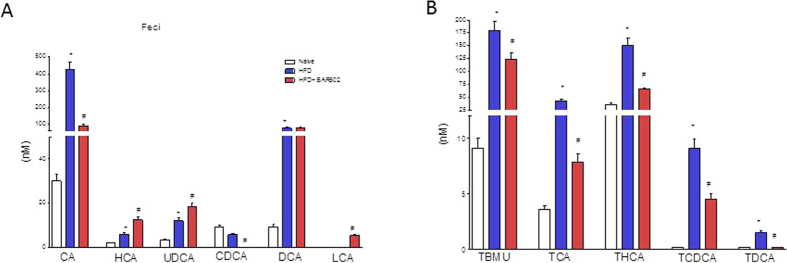
BAR502 activates FXR and reduces the synthesis of primary bile acids. Bile acid pool was assayed by measuring fecal levels of (**A**) unconjugated (CA, HCA, CDCA, UDCA, CDCA, DCA, LCA) and (**B**) conjugated (TβMCA, TCA, THCA, TCDCA, TDCA) bile acids. Results are the mean ± SE of 5 mice per group. *p < 0.05 versus naive mice, #p < 0.05 versus HFD mice.

As shown in Fig. 6A and B, feeding mice a HFD increased the fecal concentrations of nonconjugated bile acids, CA, HCA, UDCA and DCA (n = 5, p < 0.05). Moreover, the HFD fat diet significantly increased the fecal concentration of tauro(T)-conjugated bile acids (TβMCA, TCA, THCA, TCDCA and TDCA) (n = 5, p < 0.05). Administering mice with BAR502 attenuated these changes and significantly reduced the fecal concentrations of all T-conjugated bile acids (n = 5, p < 0.05) as well as that of primary bile acids, CA and CDCA, but increased the excretion of HCA, UDCA and LCA (n = 5, p < 0.05).

### BAR502 promotes the browining of preadipocytes *in vitro*

**Figure 7:**
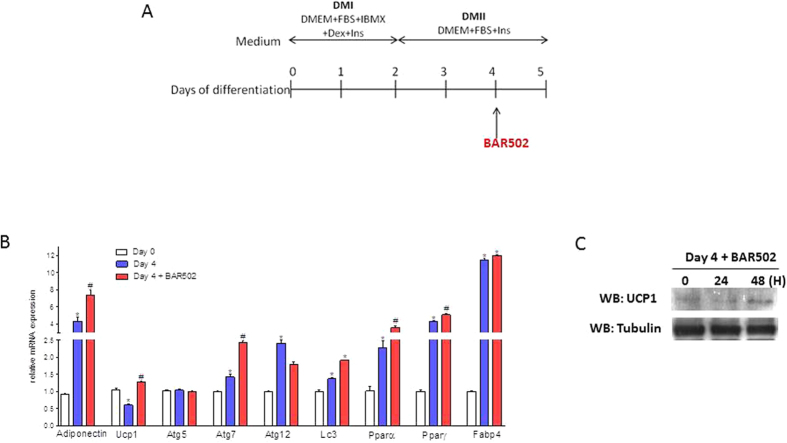
Exposure of 3T3-L1 pre-adipocytes to BAR502 increases Ucp1 expression. (**A**) 3T3-L1 pre-adipocytes were differentiated into mature white adipocytes for 4 days and then stimulated 24 hours with 10 μM BAR502. (**B**) Total RNA was extracted from cells and used to evaluated the relative mRNA expression of adipogenic marker genes, genes involved in brite differentiation and autophagic genes by RT-PCR. Results are the mean ± SE of 2 experiments. *p < 0.05 versus undifferentiated cells (Day 0); #p < 0.05 versus differentiated cells (Day 4). Values are normalized to GAPDH, the relative mRNA expression is expressed as 2^(−ΔΔCt)^. (**C**) Expression of UCP1 wa also investigated by western blot analysis. Then Western blot shows that exposure of 3T3 –L1 cells to BAR502 increases the expression of UCP1 protein. The blot shown is representative of two others showing the same pattern.

The ability of BAR502 to induce the browning of WAT, was confirmed *in vitro*. For this purpose 3T3-L1 pre-adipocytes were differentiated into mature white adipocytes for 4 days and then exposed for 24–48 hours to BAR502, 10 μM as shown in Fig. 7A. The relative mRNA expression of adipogenic genes (i.e. adiponectin, PPARα, PPARγ and FABP4) and genes involved in brite differentiation (i.e. UCP1) and autophagic genes (i.e. ATG5, ATG7, ATG12 and LC3α) was measured at the end of the study (Fig. 7B). Despite the fact that exposure to BAR502 did not reverted expression of markers of adipocytes differentiation (adiponectin, PPARα, PPARγ and FABP4 (AP2), it increased the UCP1 mRNA and protein (Fig. 7B and C) and ATG7 and LC3α mRNAs (Fig. 7B; n = 3, p < 0.05).

### BAR502 exerts anti-fibrotic activities in CCl_4_-treated mice

**Figure 8:**
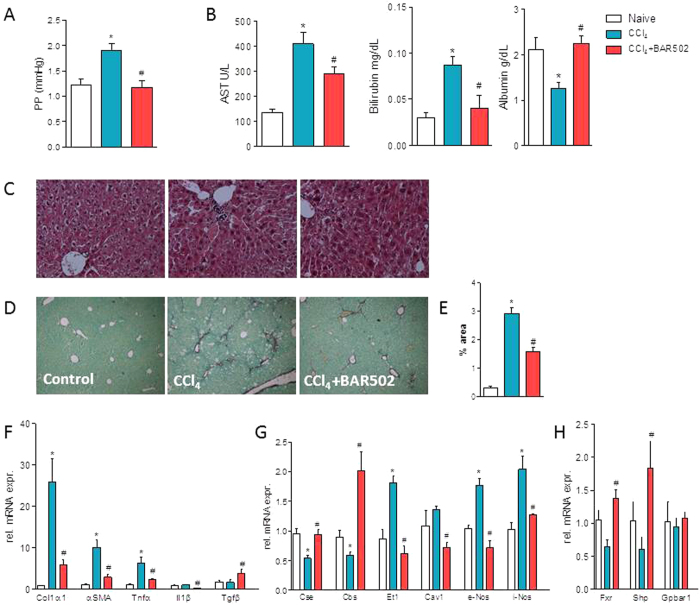
BAR502 protects against liver fibrosis induced by CCl_4_. Liver fibrosis was induced by carbon tetrachloride (CCl_4_) administration (i.p. 500 μL/Kg body weight, twice a week for 8 weeks). CCl_4_ mice were randomized to receive BAR502 (15 mg/Kg daily by gavage) or vehicle (distilled water). Data shown are (**A**) Portal pressure, (**B**) plasmatic levels of AST, Bilirubin and Albumin. (**C**) Hematoxylin and eosin (H&E) staining and (**D**) Sirius red staining on mice liver tissues; (**E**) Image J quantification of Sirius red staining. Total RNA extracted from liver was used to evaluate by RT-PCR the relative mRNA expression of (**F**) marker genes of fibrosis, (**G**) genes involved in endothelial function and (*H*) Nuclear Receptor genes. Results are the mean ± SE of 6–12 mice per group. *p < 0.05 versus naive mice. #p < 0.05 versus CCl_4_ alone.

Because fibrosis is an essential therapeutic target in liver disorders, and BAR502 reverses liver fibrosis in HFD model, we have then searched whether this compound reverts liver fibrosis in a model of severe extracellular matrix (ECM) remodeling induced by treating mice with CCl_4_. As illustrated in Fig. 8, treating mice with CCl_4_ resulted in severe liver injury as measured by assessing AST and bilirubin plasma levels, and caused a parenchymal cell extinction as indicated by the marked reduction of albumin plasma levels and increased portal pressure (Fig. 8A and B). These biochemical features associate with development of severe fibrosis as demonstrated by morphometric analysis of liver samples stained with Sirius red. The fibrotic area increased approximately 8-folds in response to CCL_4_ administration. Supporting these phenotypic changes, the liver expression of pro-fibrogenetic genes, including α1-Collagen and αSMA was increased by 10–25 folds in response to CCl_4_. Treating mice with BAR502 reversed this pattern and protected against liver fibrosis development. BAR502 reduced AST and bilirubin plasma levels, increased albumin plasma levels and reduced ECM deposition (Fig. 8), as measured by assessing the fibrosis score and expression of pro-fibrogenetic genes.

**Figure 9:**
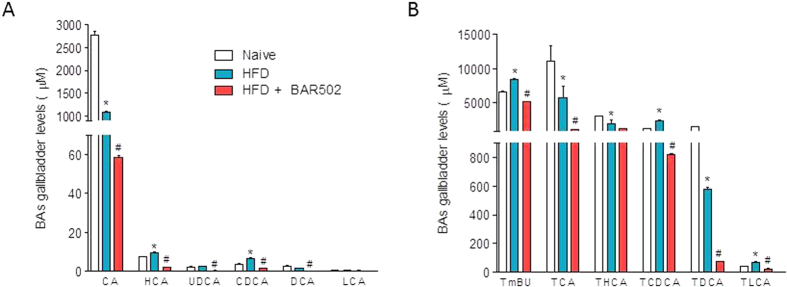
BAR502 modulates bile acid pool in CCl_4_ mice. (**A,B**) Gallbladder levels of unconjugated (CA, HCA, CDCA, UDCA, CDCA, DCA, LCA) and conjugated (TβMCA, TCA, THCA, TCDCA, TDCA) bile acids are shown. Results are the mean ± SE of 6–12 mice per group. *p < 0.05 versus naive mice. #p < 0.05 versus CCl_4_ alone.

Exposure to BAR502 also reduced portal pressure (Fig. 8A). This effect on portal pressure was linked to reduction of ECM deposition but also involved reshaping of genes such as Cse, Cbs, Et-1 and caveolin 1 which are involved in generation of endogenous vasodilators. Finally, confirming the fact that this agent is a FXR ligand, treating CCl_4_-mice with BAR502 resulted in a profound induction of Shp mRNA in the liver (Fig. 8H), and reduced the synthesis of primary acids (CA and CDCA) as shown in Fig. 9 (# denotes P < 0.05 versus CCl_4_ alone).

## Discussion

FXR and GPBAR1 are receptors for bile acids expressed in entero-hepatic tissues, adipocytes, muscles and vessels^14^,^15^,^25^,^26^,^27^. Activation of these receptors by natural and synthetic ligands reshapes host metabolism by acting on multiple checkpoints. While some of these targets are overlapping, others appear to be specific for an individual receptor. Thus while FXR activation regulates bile acid synthesis and cholesterol/triacylglycerol metabolism^28^, GPBAR1 increases energy expenditure and ameliorates insulin sensitivity^29^, suggesting that co-activation of the two receptors could be a rational approach to reduce the load of unwanted effects that might results from the selective activation of a single receptor. Thus, while activation of FXR increases the neo-glucogenesis in fasting, leading to reduce glycemic control^30^, and causes itching^18^, GPBAR1 ligand increase gallbladder weight^1^ and activate itching receptors in the skin^31^.

BAR502 is a recently discovered non-bile acid, steroidal, dual FXR and GPBAR1 ligand^20^. Here, we report that 9 weeks treatment with BAR502 reshapes the lipid metabolism and reverses the steato-hepatitis and fibrosis in mice administered a HFD. Reduction of liver cholesterol associates with a slight increase in circulating levels of HDL, indicating that in addition to inhibition of hepatic cholesterol synthesis, the main mechanism supporting the beneficial effect of BAR502 is the induction of pathways involved in cholesterol excretion from hepatocytes. Supporting this view we detected a robust reduction in the expression of FAS, a gene that encodes for the fatty acid synthase, a rate-limiting enzyme involved in fatty acid synthesis, along with an increased expression of ABCG5, a membrane transporter involved in cholesterol efflux from hepatocytes^32^. Additionally, exposure to BAR502 reduced the expression of CD36, a gene encoding for the hepatic fatty acid translocase, a membrane transporter involved in the “reverse” cholesterol transport^33^. CD36 binds long-chain fatty acids and facilitates their transport into cells, thus participating in muscle lipid utilization, adipose energy storage, and gut fat absorption and possibly contributing to the pathogenesis of metabolic disorders, such as diabetes and obesity^33^. CD36 is the quantitatively most important scavenger receptor for uptake of oxidized lipoproteins by hepatocytes and previous studies have shown that its upregulation is associated with insulin resistance, hyper-insulinemia and increased steatosis in patients with NASH^33^,^34^. Because the translocation of this fatty acid transporter to the plasma membrane of hepatocytes is thought to contribute to liver fat accumulation in patients with NAFLD^34^, its counter-regulation by BAR502 might contribute to the beneficial effects of this agent on lipid accumulation in the liver.

Analysis of FXR target genes in the liver and intestine, demonstrates that BAR502 behaves as a FXR agonist *in vivo*; indeed, in both tissues treating mice with BAR502 increased the expression of SHP, a canonical FXR targeted gene^28^. Further on, BAR502 increased the expression of FGF15^35^ in the small intestine and decreased the expression of CYP7A1 in the liver^28^. This coordinated changes are fully consistent with activation of FXR in these tissues.

FXR inhibits the synthesis of endogenous bile by overlapping mechanisms that converge in reducing the expression/activity of CYP7A1^28^. In hepatocytes, FXR negatively regulates CYP7A1 expression by activating SHP, which in its turn represses the expression of CYP7A1 by inhibiting the activity of liver receptor homolog 1 (LRH-1), an orphan nuclear receptor that is known to regulate CYP7A1 expression positively^28^. Additionally, FXR regulates CYP7A1 by SHP-independent mechanisms that requires the release of intestinal FGF15 (FGF19 in humans) which binds to the FGF receptor 4 (FGFR4) in hepatocytes and activates a signal-related kinase (ERK) and Jun N-terminal kinase (JNK) to inhibit CYP7A1 and bile acid synthesis^35^.

The fact that BAR502 represses the synthesis of endogenous bile acids (a marker of FXR activation) is confirmed by the analysis of the composition of bile acid pool. Exposure of HFD mice to BAR502 resulted in a profound reduction of the primary bile acids CA and CDCA along with their taurine conjugates (TCA and TCDCA and TβMCA). Taken together, these data demonstrate that BAR502 is a potent FXR ligand *in vivo.*

In addition, BAR502 activates GPBAR1 *in vivo* as demonstrated by increased expression of GLP1 a canonical GPBAR1 target in the intestine, and promoted energy expenditure as indicated by the significant weight loss observed in mice exposed to this agent despite mice treated with BAR502 had similar food intake in comparison with mice feed HFD alone (Fig. 1A).

The mechanism involved in energy expenditure is likely related to the ability of BAR502 to increase the activity of BAT and promoting the browning of WAT^36^,^37^. BAT is a major site of non-shivering thermogenesis in mammal^36^. BAT depots of rodents arise from mesenchymal precursor cells common to the myogenic cell lineage and are being called “classical” or “developmentally programmed” brown adipocytes. However, brown adipocytes may appear after thermogenic stimuli at anatomical sites corresponding to WAT. This process is called the “browning” of WAT^38^. The brown adipocytes appearing in WAT are indicated as beige or brite^38^. The brite/beige adipocytes are similar to BAT as they have increased mitochondrial biogenesis, multilocular lipid droplets and the expression of the BAT-specific UCP1^39^,^40^. UCP1 resides in the mitochondrial inner membrane and acts as a proton channel which dissipates proton motive force as heat instead of ATP production^39^. While there is no evidence that the thermogenic function of the beige/brite adipocytes differs from that of classical brown adipocytes it has been suggested that in rodents the browning process might protect against obesity^41^,^42^,^43^.

The epWAT is the predominant site for storage of fat in rodents with adipocytes representing the majority cell type within this tissue. We found that feeding an HFD for 18 weeks caused a three-fold increase of epWAT weight. The histopathology analysis of epWAT demonstrates that HFD hypernutrition alters the fat morphology with a dramatic increase of large-size adipocytes and influx of inflammatory cells. Treating HFD mice with BAR502 reshaped the epWAT morphology. BAR502 caused a further increase in the epWAT weight but improved the overall adipose health and, bona fide, energy expenditure by promoting the transition of epWAT toward a beige/brown phenotype as indicated by marked increase of Ucp1 expression (mRNA and immunohistochemistry). In addition to Ucp1 treatment with BAR502 increased the expression of Cited1^43^,^44^. Previous studies have shown that subcutaneous WAT is particularly prone to browning and exposure to cold or selective β-adrenergic receptor agonist induces the expression of thermogenic genes (UCP1 and PGC1α) and appearance of multilocular adipocytes in WAT depots^37^. Because the beige transition in our study was obtained in condition of thermoneutrality, it appears that BAR502 exerts its beneficial effects by promoting the browning of epWAT.

Whether these findings might have a translational readout, remains to be proved. The current interest in BAT biology largely stems from the identification of active BAT in adult humans^45^,^46^. Active BAT has been identified at discrete anatomical sites, especially in the upper trunk (ie, in cervical, supraclavicular, paravertebral, pericardial, and, to some extent, mediastinal and mesenteric areas). These BAT depots are largely composed of beige/brite cells and identified as bona fide BAT via analysis of cell morphology and expression of UCP1. The human BAT activity is inversely associated with obesity, age, and type II diabetes^46^, making this tissue an interesting therapeutic target^47^.

Since brown adipose tissue and skeletal muscles share developmental origins and brown adipocyte progenitors have been identified in human skeletal muscles^42^,^48^, we have investigated whether administration of BAR502 effectively induced the browning of the muscle fat. Low levels of *UCP1* mRNA were identified in mice muscle biopsies^42^,^43^, and importantly we found a robust induction of this gene in response to BAR502. Similar finding were observed for UCP2 and UCP3. These findings were consistent with the acquisition of a brown adipose phenotype in muscle of mice fed a HFD plus the dual FXR/GPBAR1 agonist.

To confirm these findings we have then investigated whether exposure to BAR502 induces a transdiffentiation of 3T3-L1 white preadipocytes into a beige phenotype *in vitro* by assessing UCP1 as a marker of browning. A significant expression of UCP1 (mRNA and protein) was detected in the control 3T3-L1 adipocytes and while mRNA expression declined during the differentiation process, exposure of cells to BAR502 resulted in recovery of UCP1 expression. Importantly, BAR502 had no effect on markers of adipocytes maturation including apinectine, FABP4, PPARα and γ. Because exposure of target cells to BAR502 increases intracellular concentrations of cAMP, these data are consistent with the fact that both IBMX (a phosphodiesterase inhibitor) and β-adrenergic receptor agonists increases UCP1 expression in 3T3-L1 cells *in vitro*^49^.

In addition to promoting cholesterol efflux and epWAT browning, the treatment with BAR502 reduced liver fibrosis. Liver fibrosis is an essential pathology component of human NASH^7^,^8^. We have previously shown that FXR agonism results in a SHP-dependent downregulation of collagen synthesis^21^. Because liver myo-fibroblasts do not express GPBAR1, the reduced deposition of ECM observed in this study is likely to reflect the activation of FXR^50^.

Because the severity of liver fibrosis is a biomarker of liver injury progression and predicts a poor prognosis in patients with NASH^8^, we have investigated whether BAR502 reduced liver fibrosis in another model of liver injury caused by exposure to CCl_4_^21^. BAR502 prevented parenchymal cell extinction as demonstrated by recovery of albumin plasma levels (a marker of hepatic biosynthetic activity), reduced the fibrosis score, and expression of genes encoding for known biomarkers of ECM deposition, including αSMA and α1-Collagen. Additionally, BAR502 increased the expression of SHP, a marker of FXR activation in the liver, and a negative regulator of HSC activation^21^

One important consequence of intense fibrogenesis caused by CCl_4_ is the development of portal hypertension, a life-threatening complication that occurs in late-stage cirrhosis^50^. Treating mice with BAR502 protected against development of increased portal pressure caused by CCl_4_ administration. This protection was the result of both a reduction of liver fibrogenesis and induction of eNOS and CSE, two genes involved in the production of nitric oxide (NO) and hydrogen sulfide (H_2_S). Because eNOS and CSE are expressed by liver sinusoidal cells and these cells express GPBAR1, these effects could be considered a biomarker of GPBAR1 activation in the liver^50^,^51^.

In summary, we have shown that BAR502, a dual FXR and GPBAR1 ligand, confers protective effects against hepatic lipid partitioning in a model of NASH induced in mice by a HFD. These beneficial effects appear to be consequences of improved adipose function with browning of WAT. Present data suggest that dual FXR/GPBAR1 ligand might have utility in the treatment of NASH.

## Additional Information

**How to cite this article:** Carino, A. *et al*. BAR502, a dual FXR and GPBAR1 agonist, promotes browning of white adipose tissue and reverses liver steatosis and fibrosis. *Sci. Rep.* **7**, 42801; doi: 10.1038/srep42801 (2017).

**Publisher's note:** Springer Nature remains neutral with regard to jurisdictional claims in published maps and institutional affiliations.
